# Ironing out the distribution of [2Fe-2S] motifs in ferrochelatases

**DOI:** 10.1016/j.jbc.2021.101017

**Published:** 2021-09-25

**Authors:** R. Sophia Weerth, Amy E. Medlock, Harry A. Dailey

**Affiliations:** 1 Department of Microbiology, University of Georgia, Athens, Georgia, USA; 2 Department of Biochemistry & Molecular Biology, University of Georgia, Athens, Georgia, USA; 3 Augusta University/University of Georgia Medical Partnership, Athens, Georgia, USA

**Keywords:** heme, iron-sulfur protein, bioinformatics, evolution, porphyrin, iron metabolism, AHB, alternate heme biosynthetic, CAB, chlorophyll a/b binding, CDP, coproporphyrin-dependent pathway, EPP, erythropoietic protoporphyria, MCF, motif-containing ferrochelatase, PDP, protoporphyrin-dependent pathway

## Abstract

Heme, a near ubiquitous cofactor, is synthesized by most organisms. The essential step of insertion of iron into the porphyrin macrocycle is mediated by the enzyme ferrochelatase. Several ferrochelatases have been characterized, and it has been experimentally shown that a fraction of them contain [2Fe-2S] clusters. It has been suggested that all metazoan ferrochelatases have such clusters, but among bacteria, these clusters have been most commonly identified in Actinobacteria and a few other bacteria. Despite this, the function of the [2Fe-2S] cluster remains undefined. With the large number of sequenced genomes currently available, we comprehensively assessed the distribution of putative [2Fe-2S] clusters throughout the ferrochelatase protein family. We discovered that while rare within the bacterial ferrochelatase family, this cluster is prevalent in a subset of phyla. Of note is that genomic data show that the cluster is not common in Actinobacteria, as is currently thought based on the small number of actinobacterial ferrochelatases experimentally examined. With available physiological data for each genome included, we identified a correlation between the presence of the microbial cluster and aerobic metabolism. Additionally, our analysis suggests that Firmicute ferrochelatases are the most ancient and evolutionarily preceded the Alphaproteobacterial precursor to eukaryotic mitochondria. These findings shed light on distribution and evolution of the [2Fe-2S] cluster in ferrochelatases and will aid in determining the function of the cluster in heme synthesis.

Heme synthesis is an evolutionarily old biosynthetic pathway. Heme, as a cofactor, is used in the vast majority of identified life forms, and most organisms that use heme possess their own biosynthetic pathway to produce it. As a result, the enzymes required for heme synthesis are highly conserved (1, 2). One of these enzymes, ferrochelatase, is a member of the class II metal chelatase family and is typically monomeric or homodimeric. *In vivo* ferrochelatase catalyzes the insertion of divalent iron into the porphyrin ring during heme biosynthesis, although the enzyme has some promiscuity with other metal and porphyrin substrates *in vitro* (1, 3). Ferrochelatase is less promiscuous *in vivo* as alternate porphyrins are not present in the cell, and other bioavailable divalent metals are either of low abundance or not delivered to ferrochelatase for insertion.

The ferrochelatase reaction is an important regulatory node for heme biosynthesis existing at the convergence of porphyrin synthesis and iron supply. Ferrochelatase activity, and hence heme biosynthesis, is dependent upon iron availability and is, therefore, *de facto* regulated by iron. Studies of select bacterial systems have also shown that ferrochelatase activity is an indicator used by the cell to regulate iron homeostasis through the iron response regulator (porphyrin synthesis being among this) (4). In addition, within photosynthetic organisms, ferrochelatase is downregulated to allow the porphyrin to be metalated by magnesium chelatase for chlorophyll biosynthesis (5, 6). Ferrochelatase is also of clinical relevance, as low activity in humans causes the condition of erythropoietic protoporphyria (EPP) (7).

To date, three known heme synthesis pathways have been elucidated that use a ferrochelatase; the protoporphyrin-dependent pathway (PDP), found in gram-negative bacteria and eukaryotes, the coproporphyrin-dependent pathway (CDP) found in Actinobacteria and Firmicutes, and the alternate heme biosynthetic (AHB) pathway found in archaea (1). While ferrochelatase is found in all three pathways, in each the enzyme metalates a different substrate (Fig. 1). In the PDP ferrochelatase inserts iron into protoporphyrin IX, forming protoheme IX, the final product of the pathway, and is therefore specified as protoporphyrin ferrochelatase or PpfC (1). In the CDP ferrochelatase inserts iron into coproporphyrin III forming coproheme III, specifying the enzyme as coproporphyrin ferrochelatase or CpfC. This reaction forms the penultimate product, which is then decarboxylated to form protoheme (8). In the AHB pathway ferrochelatase metalates sirohydrochlorin forming siroheme, specifying it as sirohydrochlorin ferrochelatase or SirB (9). Although the AHB pathway is specific to archaea, siroheme is also an intermediate in heme *d*_1_ synthesis and a cofactor required in sulfur and nitrogen metabolism (10, 11, 12, 13). Therefore, sirohydrochlorin ferrochelatase is an enzyme found frequently in prokaryotes and some fungi, including in species lacking the AHB pathway. Of note is that sirohydrochlorin ferrochelatase shows higher structural similarity to cobalt chelatase CbiX, another class II metal chelatase involved in cobalamin (B_12_) synthesis, than it does to PpfC and CpfC (9). The current study focuses on PpfC and CpfC and does not include sirohydrochlorin ferrochelatase.

**Figure 1:**
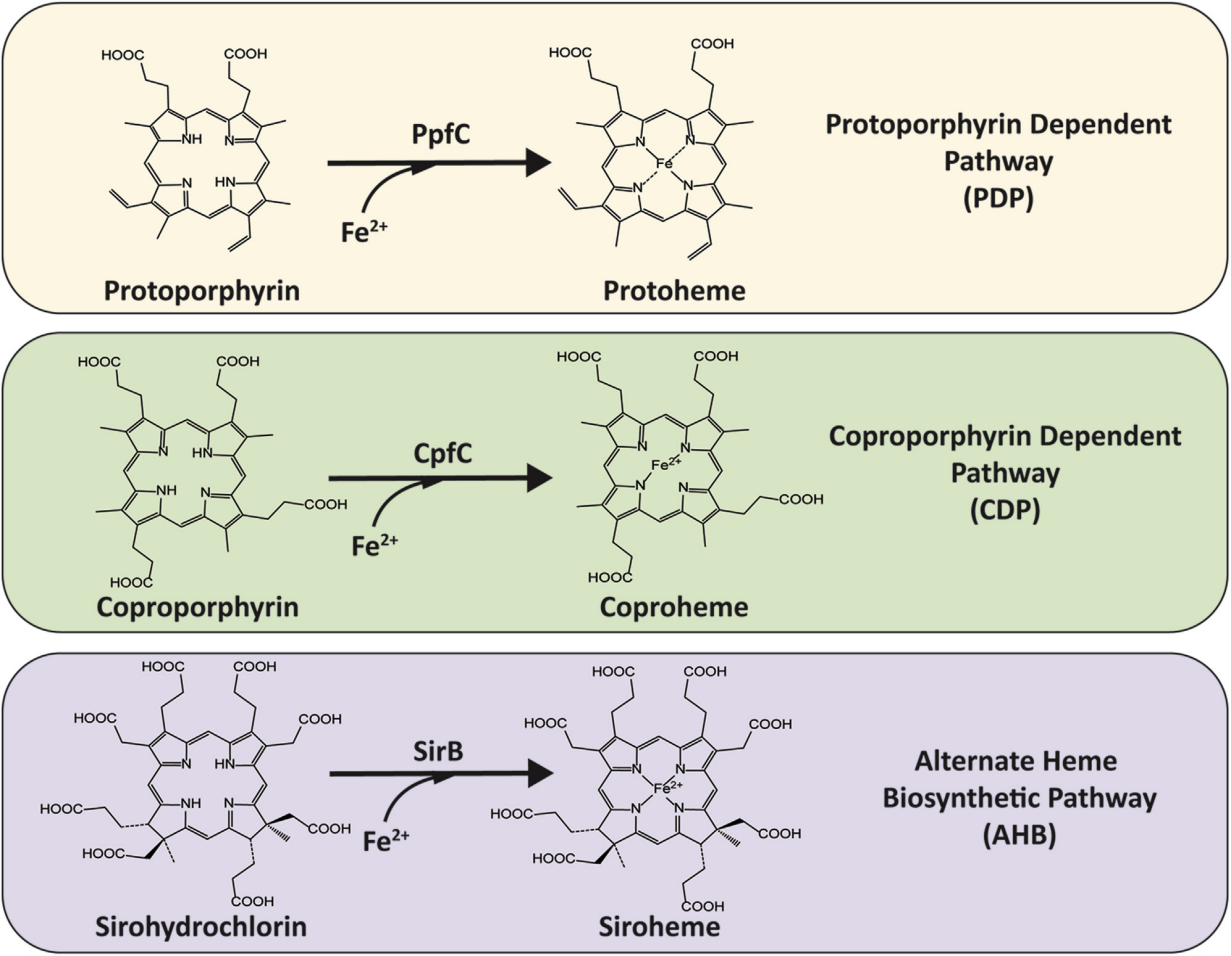
**Ferrochelatase activity.** The panels show ferrochelatase activity from the three biosynthetic pathways (as labeled). The abbreviations above the arrows indicate the specific Ferrochelatase enzyme; protoporphyrin ferrochelatase (PpfC), coproporphyrin ferrochelatase (CpfC), and sirohydrochlorin ferrochelatase (SirB). The chemical structures denote the substrates and products of the enzymes (as labeled).

Ferrochelatases have been found with and without [2Fe-2S] clusters, a feature whose purpose has remained elusive for the past 27 years. Following the first description of ferrochelatase activity in 1955 by Goldberg's group, a variety of studies in diverse organisms were conducted on the subject (14). However, it was not until 1994, with the expression and characterization of recombinant human ferrochelatase, that a [2Fe-2S] cluster was identified as part of a ferrochelatase protein (15). Prior purified ferrochelatases, including those of *Spirillum itersonii* and *Saccharomyces cerevisiae*, did not contain a [2Fe-2S] cluster (16, 17). Over the past two decades, a dozen additional [2Fe-2S]-containing ferrochelatases have been confirmed experimentally, and available genome sequence data are consistent with the cluster being present in all metazoan ferrochelatases and a number of bacterial ferrochelatases (3, 18, 19, 20). However, the purpose of the [2Fe-2S] in ferrochelatase is still elusive. Biochemical analyses of the [2Fe-2S] clusters in ferrochelatase revealed three things. First, microbial ferrochelatase [2Fe-2S] clusters are significantly more stable than mammalian clusters. Specifically, clusters contained in bacterial ferrochelatases remain intact when stored in 1% SDS at room temperature, whereas mammalian ferrochelatase clusters are oxygen- and temperature-labile and sensitive to NO (19, 21). Second, the clusters' redox ability is not essential for activity, but may play a role in modulating enzyme activity, at least in higher metazoans (21, 22). For bacteria, the role of the cluster is less certain since elimination of the cluster through the substitution of the [2Fe-2S] coordinating cysteines with serines does not eliminate enzyme activity as is found with the metazoan enzyme (23, 24). Indeed, these Cys to Ser variants complement an *Escherichia coli* ferrochelatase knockout strain in trans and showed similar K_m_s as the wild-type enzyme when heterologously expressed (19, 20). Third, the cysteine coordinating residues can be found in one of two locations within the sequence: within an internal insertion sequence or at the C-terminal extension (Fig. 2*A*) (20). Interestingly structural modeling of cluster-containing bacterial ferrochelatases onto the known crystal structure of ferrochelatase with the C-terminal cluster demonstrates that the spatial positioning of the cluster is likely to be highly similar between the two motifs (Fig. 2*B*) (1, 25). So while the functional role of the [2Fe-2S] cluster is still unknown, its prevalence within ferrochelatase sequences is noteworthy and indicates its potential necessity for either a common ancestor or regulatory purposes.

**Figure 2:**
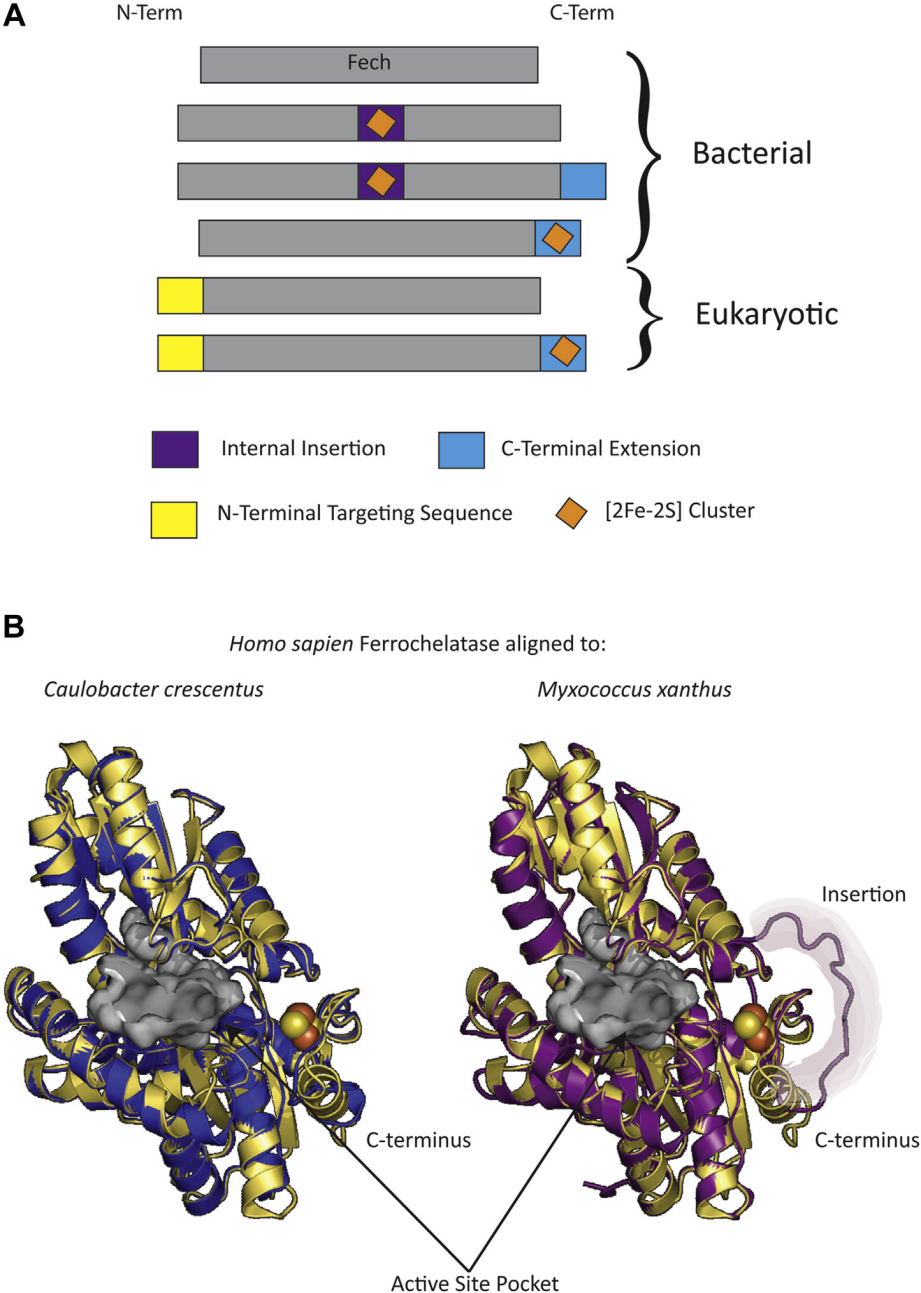
**Models of bacterial ferrochelatases.***A*, simplified model of ferrochelatase sequence diversity and cluster placement. The *gray bar* indicates the ferrochelatase core protein, and the *colored boxes* indicate the N-terminal targeting sequence (*yellow*), the internal insertion (*purple*), and the C-terminal extension (*blue*). *Orange diamonds* indicate the placement of the [2Fe-2S]. *B*, above are structural models (rendered by I-Tasser), in *blue* and *purple*, that were aligned and overlayed to human ferrochelatase (PDB 1hrk) in *yellow*. The *left cartoon* shows the structural model of *Caulobacter crescentus* ferrochelatase (*blue*), and the *right* shows the structural model *Myxococcus xanthus* ferrochelatase (*purple*). The active site pocket of 1hrk was displayed as a gray surface representation and the [2Fe-2S] cluster of 1hrk was displayed as *yellow* and *orange* spheres. Labels identify the C-terminus in both alignments, and the internal insertion sequence was highlighted and labeled in the right alignment.

When the presence/absence of [2Fe-2S] cluster in ferrochelatases was first assessed, only a limited number of sequenced genomes were available (1, 3, 19, 20). As current models for cluster distribution among bacteria result from experimental studies on a limited number of enzymes, and because identification of putative cluster containing ferrochelatases can be based upon simple sequence motifs, we undertook a genomic study to determine the distribution and diversity of [2Fe-2S] clusters in available genomes. With the recent increased availability of sequenced genomes, it has become possible to more fully examine the prevalence of the [2Fe-2S] cluster in the microbial community. With the use of the Joint Genome Institute Integrated Microbial Genome (IMG.JGI) database, we show that [2Fe-2S] clusters are not highly prevalent among known microorganisms. However, for those organisms that do possess a cluster, we have identified the position of the coordinating cysteine residues, delineating whether the cluster is coordinated by a set of four cysteines present in an internal motif or in the C-terminal extension. In addition, we examined select properties of organisms possessing a cluster containing ferrochelatase to find if there is an obvious relationship between the presence of a cluster and the habitat of the organism.

## Results

### Ferrochelatase-containing species

A total of 9644 completed genomes were analyzed using the IMG.JGI online portal and found to possess genes encoding for a putative ferrochelatase (E.C. 4.99.1.1) (26, 27). Among these genomes are 75 eukaryotic and 9569 bacterial species. Archaeal genomes were not included in this analysis since, as mentioned above, archaeal ferrochelatases are unique from bacterial and eukaryotic ferrochelatases. In addition, the presence of the archaeal ferrochelatase does not indicate presence of heme prototrophy.

To avoid bias from oversampling, the genome data sets were pared down to 418. Within these genomes, 497 putative ferrochelatase encoding ORFs were identified. The number of putative ferrochelatase genes is higher than the number of genomes since 69 possess two copies of the putative ferrochelatase gene, one (*Cyanobium gracile* PCC 6307) contains three copies and one (*Zea mays* cv. B73) contains seven copies.

### Ferrochelatase verification

Some species, such as the heme auxotrophic *Caenorhabditis elegans*, encode nonfunctional ferrochelatase, so those were eliminated from our data set in the following fashion (28). Enzymatic and structural analyses have identified a histidine residue within the active site that is essential for enzyme activity. This histidine is responsible for proton abstraction from the porphyrin ring to allow metalation (29). Sequences lacking this residue will not encode a functional enzyme and were eliminated from further analysis. In total 14 sequences were found to be missing the catalytically essential histidine and were eliminated from the final sequence list. Of the inactive ferrochelatase encoding sequences, 11 were dysfunctional duplicate copies in organisms that possessed a second functional ferrochelatase. Interestingly the fungal pathogen *Botrytis cinerea* (genome ID: 645666404) encodes two putative ferrochelatase ORFs that, if the published sequences are correct, both encode a nonfunctional enzyme. This organism is typically cultured without exogenous heme, indicating that its genome likely encodes a functional ferrochelatase (30). In addition, *B. cinerea* possesses two copies of hydroxymethylbilane synthase (EC:2.5.1.61), an upstream enzyme in porphyrin metabolism, and one copy of coproporphyrinogen oxidase (EC:1.3.3.3) and protoporphyrinogen oxidase (EC:1.3.3.4) each, indicating that *B. cinerea* B05.10 possesses the capability to synthesize porphyrin precursors for heme synthesis. Ultimately, we identified 482 putative functional ferrochelatases from the 414 genomes examined (Table S1).

### [2Fe-2S] motifs

**Figure 3:**
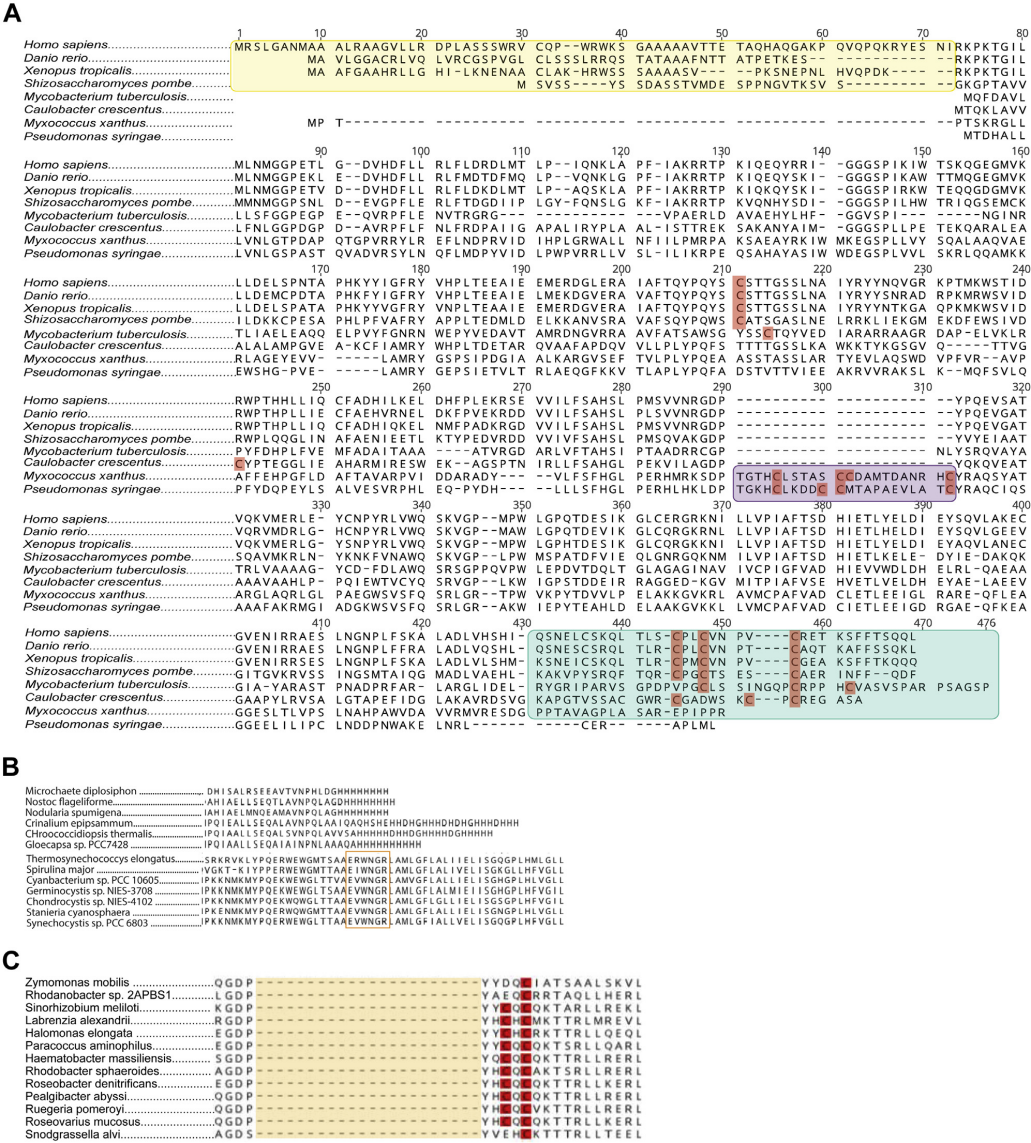
**Ferrochelatase amino acid sequence alignment.***A*, an alignment of eight ferrochelatase sequences confirmed to coordinate a [2Fe-2S] cluster. The following sequence attributes were highlighted accordingly: N-terminal extension (*yellow*), internal insertion (*purple*), C-terminal tail (*cyan*), [2Fe-2S] coordinating cysteine residues (*red*). *B*, excerpts of full ferrochelatase alignment showing Cyanobacterial histidine rich C-termini (*top panel*) in comparison to C-termini with CAB domains. The *orange box* highlights conserved the CAB domain motif. *C*, excerpts of full ferrochelatase alignment showing sequences that carry the internal CXC grouping. The area highlighted in *orange* is where other sequences carry the internal insertion.

After elimination of the noncatalytic putative ferrochelatases, the remaining set of 482 predicted amino acid sequences were assessed and aligned. These sequences vary greatly in length from 270 to 683 amino acids. The outlier in this group, the 683 amino acid ferrochelatase of *Cutibacterium acnes*, has been experimentally demonstrated to be a fusion protein that contains both the ferrochelatase and the coproheme decarboxylase (ChdC) enzyme (31). For inclusion in the alignment, the ChdC portion of the sequence was eliminated resulting in a 448 amino acid length protein. The variation in sequence length has been noted before, as ferrochelatases from different species can contain an N-terminal extension (an organellar targeting sequence in eukaryotes), an internal 22–34 amino acid insertion and/or a C-terminal tail (Fig. 3*A*) (3, 17). From our data set 56 ferrochelatase sequences contain an N-terminal extension, 63 contain the internal insertion, and 215 contain a C-terminal tail.

During the assessment of the ferrochelatase C-termini, a subset of cyanobacterial ferrochelatase, were distinct. These sequences differed significantly from other C-terminal extensions, as they are histidine-rich (Fig. 3*B*). In some other cyanobacteria, there is a conserved C-terminal EXXNXR motif as part of a chlorophyll *a*/*b* binding (CAB) domain, which is required for activity in those species (32). It was observed that the presence of the CAB domain and EXXNXR was mutually exclusive. The second subset of CAB-less cyanobacterial ferrochelatases have not been previously identified or studied.

To generate a consensus [2Fe-2S] motif, the [2Fe-2S] cluster motifs from eight characterized ferrochelatases were assessed: *Homo sapiens* (gene ID 639337888)*, Mycobacterium tuberculosis* (gene ID 2633794335)*, Schizosaccharomyces pombe* (gene ID 638217021), *Myxococcus xanthus* (Gene ID 638022924), *Caulobacter crescentus* (Gene ID: 637089825), *Pseudomonas syringae* (gene ID 637651310), *Danio rerio* (Gene ID: 639505885), and *Xenopus tropicalis* (Gene ID 2507956732) (3, 18, 20). Evaluation of the [2Fe-2S] coordinating cysteines in these ferrochelatase sequences led to the following motif using PROSITE specifications, **C**X(1,11)**C**X(1,11)**C**, where the numbers indicate the outer bounds of the residue spacing (33). The motif relies on the grouping of three cysteines, as the fourth cysteine is spaced too distant in some proteins to provide a reliable 4 cysteine motif. However, some sequences do have all four cysteines positioned closely together, due to a XCCX grouping commonly found in the internal insertion. It has been demonstrated that both of these adjacent cysteines are involved in [2Fe-2S] coordination (20). To allow for the consensus motif to include the motifs with the XCCX grouping but not exclude motifs that possess a CXC grouping (shown to be part of other [2Fe-2S] clusters), the CX(1,11) annotation was included, where X allows for any amino acid at position one, including cysteine. As a result, the sequences with a motif carrying the XCCX grouping have all 4 [2Fe-2S] coordinating cysteines within the motif even though the consensus motif does not require it.

Cysteine groupings are common in ferrochelatase sequences. A subset of ferrochelatases lacking the internal insertion and the C-terminal extension carry an internal CXC grouping instead (Fig. 3*C*). *Ruegeria pomeroyi* ferrochelatase carries this CXC grouping and structural modeling indicates that the cysteines are located at the homodimer interface. Preliminary investigation into the purpose of these cysteines has shown that the heterologously expressed protein is a dimer and purifies with a tightly bound red pigment. This pigment is likely a nonmetalated porphyrin as the protein fluoresces and the metal content is only 1–10% of the protein concentration. It is possible that this pigment is coordinated at the homodimer interface by four cysteines (two cysteines contributed by each monomer), as when the dimer is disrupted, the pigment is lost (Weerth and Dailey, unpublished).

**Figure 4:**
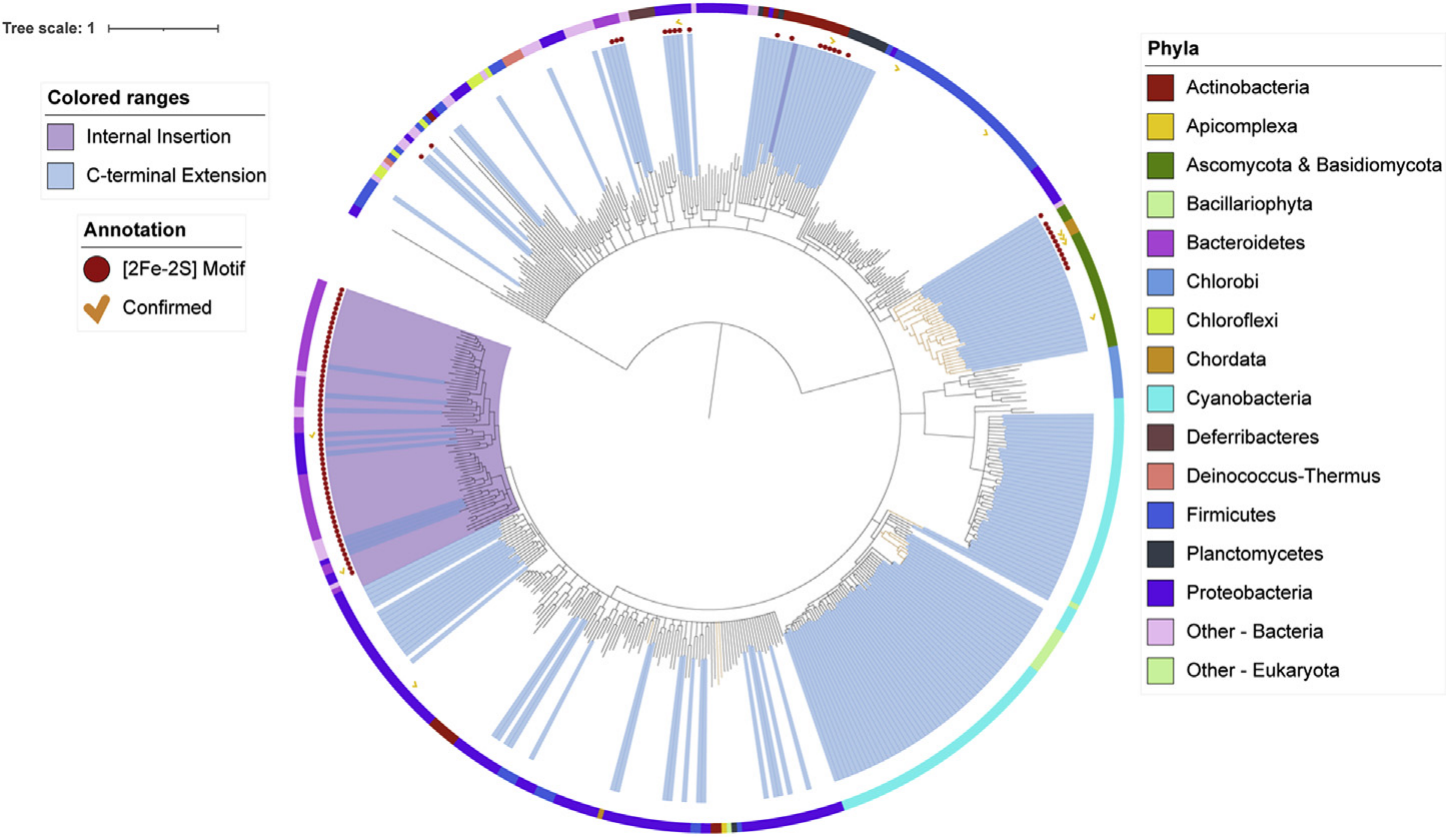
P**hylogenetic tree of ferrochelatase alignment.***Black lines* indicate bacterial species, and *orange-colored lines* indicate eukaryotic species. *Colored bars* indicate their phylum (see Phyla legend). The icons indicate the presence of the [2Fe-2S] Motif (*red dot*) and whether the enzyme has been characterized (*yellow checkmark*). The interactive version of the tree can be accessed at https://itol.embl.de/shared/1YFbOwXl23wtM.

After assessing the 482 sequences, 89 species were identified to possess a ferrochelatase that contains the **C**X(1,11)**C**X(1,11)**C** motif. These include the sequences experimentally confirmed as having a [2Fe-2S] cluster and sequences that had been previously noted as putatively [2Fe-2S] carrying ferrochelatases (3). Of the 482 putative ferrochelatase sequences, 63 have an insertion sequence, but of these only 60 contain the complete motif (Fig. 4). Three have the insertion but do not have a motif. In total, 29 of the 89 sequences have a motif located in the C-terminus. An additional 18 sequences contained a motif, but it was located in other areas of the protein. Since [2Fe-2S] clusters have only been experimentally confirmed in the insertion and the C-terminus of ferrochelatases, these 18 sequences were assessed as regular/nonmotif sequences.

Despite the alternate positioning of the motif within the sequences (internal insertion *versus* C-terminus), structural modeling suggests that the motif exists in similar areas of the protein (Fig. 2) (20). The ferrochelatase structures of *C. crescentus*, a C-terminal motif, and *M. xanthus*, an insertion containing motif, were modeled with the online server I-TASSER and aligned with the ferrochelatase crystal structure of *H. sapiens* (PDB code: 1HRK) (25, 34, 35). *H. sapiens* (human) ferrochelatase carries the [2Fe-2S] motif in the C-terminus. Aligning *C. crescentus f*errochelatase (C-terminal motif) (C-score: 1.45) with human ferrochelatase resulted in an RMSD of 0.248 and aligning *M. xanthus* ferrochelatase (insertion motif) (C-score: -0.68) with human ferrochelatase gave a RMSD of 0.249. In the *C. crescentus* alignment, the C-terminal motif surrounds the modeled [2Fe-2S] similarly to the human ferrochelatase. However, in the *M. xanthus* alignment, the insertion sequence is a disordered loop positioned around the human C-terminal but at a greater distance (Fig. 2*B*). Coordination of the *M. xanthus* [2Fe-2S] cluster by the four cysteine residues of the internal motif would restrict and compact the predicted disordered segment making it probable that the insertion sequence-coordinated cluster would exist in the same region as occupied by the [2Fe-2S] cluster in human enzyme.

### Cysteine and motif frequency

**Figure 5:**
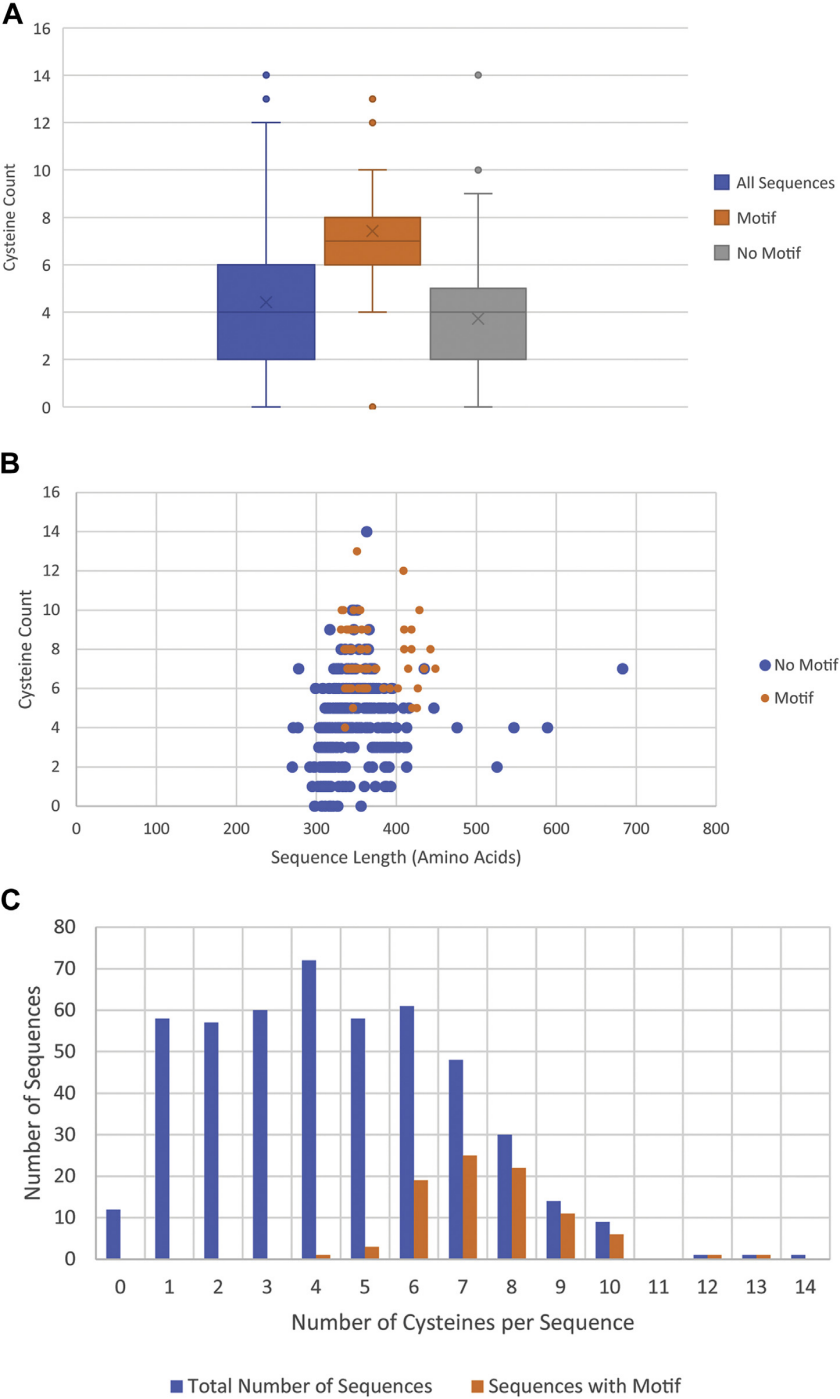
**Cysteine distribution in ferrochelatase.***A*, box and whiskers plot of cysteine count in ferrochelatase sequences. Cysteine count of all sequences (*blue*), cysteine count of sequences containing a motif (*orange*), cysteine count of sequences lacking a motif (*gray*). The arithmetic mean is represented by the X within each box, and the *dots* represent the outliers. *B*, a scatter plot depicting the sequence length and cysteine quantity for each sequence. Sequences with a motif are represented by *orange dots*, and sequences without a motif are represented by *blue dots*. *C*, a bar graph showing the number of sequence count by cysteines per sequence. *Blue bars* depict the total number of sequences, and orange bars depict the sequences with motifs.

The cysteine frequency varies greatly between ferrochelatase sequences ranging from 0 to 14 cysteines (0–3.9% of the sequence length), with an average mean of 4.4 cysteines per sequence (Fig. 5*A*). However, there is no direct correlation between total protein amino acid sequence length and cysteine frequency (Fig. 5*B*). Additionally, the presence of a high number of cysteines in a ferrochelatase sequence does not guarantee the presence of a motif. For example, ferrochelatase of the malaria parasite *Plasmodium falciparum* (Genome ID: 2802429542 Gene ID: 2805692653) contains the highest number of cysteines (14 cysteines), but does not contain a motif and there has been no evidence of it containing an iron sulfur cluster (36). However, our analysis shows that there is an increased likelihood of a motif being present in sequences containing six to ten cysteines (Fig. 5*C*). For the sequences that contain six to ten cysteines, the likelihood that a sequence will contain a motif is 0.51 (±0.04), whereas the likelihood to contain a motif in sequences with four to six cysteines drops to 0.03 (±0.02). Containing more than ten cysteines has a higher probability of 0.67 but due to the low n of 3 the standard error is high at ± 0.27. A binary logistical regression models the odds ratio of containing a motif at 2.61 with a lower confidence interval of 2.13. This means that the probability of containing a motif increases by a factor of 2.61 when the number of cysteines increases by 1. As a result, containing fewer than seven or more than ten cysteines decrease the likelihood of the presence of a motif.

### [2Fe-2S] motif phylogenetic distribution

The [2Fe-2S] motifs were found in 13 of the 30 sampled phyla. However, the frequency of the motif within each phylum varies widely (Table 1, Table S2). The Bacteroidetes phylum contains the most [2Fe-2S] motif-containing ferrochelatase sequences with 44. This represents not only the greatest number of motifs, but also the highest percent of motif-containing ferrochelatases (MCFs) per phyla at 96%. The majority of the Bacteroidetes sequences contain the internal insertion, and most of the internal insertions carry the motif. However, there are two sequences that lack the internal sequence and carry the motif in the C-terminus. The two sequences that contain the motif in the C-terminus are from *Rhodothermus marinus* (Genome ID: 646311949, Gene ID: 646410483) and *Salinibacter ruber* (Genome ID: 2561511240, Gene ID: 2562594983). Interestingly the *S. ruber* genome also carries a second ferrochelatase sequence (Gene ID: 2562594010) that lacks both the motif and the internal insertion sequence. Both of the *S. ruber* ferrochelatase sequences contain the catalytic His and are assumed to be functional. The Bacteroidetes sequence that has the internal insertion but lacks a motif is from the single ferrochelatase of *Porphyromonas gingivalis* (Genome ID: 2843222565, Gene ID: 2843222810). Notably, the insertion in the *P. gingivalis* sequence has a CX(9)CC grouping indicating a partial motif. In addition, the *P. gingivalis* sequence was one of the 18 sequences that was excluded as an MCF, as the motif that was found is contained in between the internal insertion and the C-terminal extension (which did not include the CX(9)CC grouping).

**Table 1:** Motif-containing sequences by phylum

Phylum	Number of sequences	Motif-containing ferrochelatases	Percent of sequences	Insertion
Bacteroidetes	46	44	96%	43
Proteobacteria	144	16	11%	11
Actinobacteria	24	6	25%	0
Ascomycota	23	6	26%	0
Verrucomicrobia	4	4	100%	4
Chordata	4	3	75%	0
Basidiomycota	3	2	67%	0
Chlamydiae	4	2	50%	2
Planctomycetes	11	2	18%	0
Gemmatimonadetes	2	1	50%	0
Ignavibacteriae	2	1	50%	1
Nitrospirae	3	1	33%	0
Spirochaetes	4	1	25%	0

The insertion column indicates the number of sequences that contain an internal insertion sequence. Not all insertions carry a [2Fe-2S] motif. This table only includes phyla with species that contain Ferrochelatases with a [2Fe-2S] motif. Table S2 shows the complete table with all ferrochelatase sequences and further breakdown by class, order, and family.

Proteobacterial genome sequences have the second highest number of [2Fe-2S] motifs, with 16 MCFs. Specifically, five sequences contain the motif in the C-terminus and 11 in the internal insertion. However, it should be noted that MCFs are only 11% of the proteobacterial sequences assessed. Notably, this small percentage of MCFs are not clustered but rather scattered throughout the phyla. Specifically, the Acidithiobacillia and Alphaproteobacteria classes contain the sequences with a C-terminal motif. The Betaproteobacteria, Deltaproteobacteria, Gammaproteobacteria, and Oligoflexia classes contain sequences with the insertion motif. *M. xanthus*, a Deltaproteobacterium, has been confirmed to contain an [2Fe-2S] cluster in the internal insertion sequence, and *Caulobacter cresecentus* an alphaproteobacterium has been shown to have a C-terminal [2Fe-2S] cluster (20).

Actinobacteria and Ascomycota phyla each have six MCFs, which are also C-terminal motifs. Approximately one-quarter of each phyla, 25% and 26%, respectively, have MCFs. Actinobacterial MCFs are all found within the Actinobacterial class but are somewhat scattered within that class. Specifically, the Corynebacteriales, Micrococcales, Propionibacteriales, and Streptomycetales orders contain MCFs. For Ascomycota, however, the MCFs are found within all classes except for Saccharomycetes and Leotiomycetes. It is of note, that *S. pombe* from the Schizosaccharomycetes class has been confirmed to contain a [2Fe-2S] while *Saccharomyces cerevisiae* of the Saccharomycete class has been confirmed to lack the [2Fe-2S] cluster (17, 19).

Verrucomicrobia have four MCFs, all insertion motifs. The motif frequency for Verrucomicrobia is 100%; however, only four sequences were assessed for that phyla. The four MCFs are found within the Opitutaceae and Puniceicoccaceae family (three and one MCFs, respectively) as part of the Opitutae order. More sequence data are required to determine whether the motif presence is significant or due to sampling bias.

The phyla Basidiomycota, Chlamydiae, Nitrospirae, Ignavibacteriae, Gemmatimonadetes, and Spirochaetes have three or fewer MCFs. All MCFs are C-terminal, except for Chlamydiae and Ignavibacteriae, which have insertion motifs. However, like Verrucomicrobia, only four or fewer sequences were assessed for each of the phyla. Similar to Verrucomicrobia, a broader data set is required to draw meaningful conclusions.

All Chordata species, three species, sampled have also been identified as MCF containing. This is in congruence with previously published findings that all metazoan ferrochelatases are predicted to carry a [2Fe-2S] cluster (2). The [2Fe-2S] cluster containing ferrochelatase identified here, specifically that of *D. rerio* (Genome ID: 639469800, Gene ID: 639505885), *H. sapiens* (Genome ID: 639332100, Gene ID: 639337888), and *X. tropicalis* (Genome ID: 2507525015, Gene ID: 2507956732) have been confirmed to contain [2Fe-2S] clusters (15, 18, 22). However, this study identified a second ferrochelatase sequence within the *X. tropicalis* genome. This duplicate ferrochelatase of *X. tropicalis* lacks the [2Fe-2S] motif, but carries the catalytic histidine and is, therefore, likely active. Therefore, the possibility of other metazoans containing active ferrochelatases without [2Fe-2S] clusters exists, which was not previously recognized.

The Planctomycetes phylum contains two MCFs out of 11 assessed sequences, namely *Fuerstia marisgermanicae* (Genome ID: 2751185673, Gene ID: 2752837047) and *Leptospirillum ferrooxidans* (Genome ID: 2540341086, Gene ID: 2540578217). Both carry the motif in the C-terminus. Interestingly, some Planktomycetes, specifically those within the Isosphaerales, Pirellulales, and Planctomycetales orders, have cysteine rich C-termini with CX(3)CC groupings; however, a fourth cysteine to complete these motifs is missing.

Overall the phylogenetic tree (Fig. 4) of ferrochelatase sequences reveals that MCFs with the internal insertion sequence are more closely related than MCFs with the motif in the C-terminal extension. The insertion MCFs all cluster within one large clade. Within this group are also two sequences that contain the insertion sequence but lack the [2Fe-2S] motif. It is of note that one ferrochelatase of Actinobacterium *Mobiluncus curtsii* (Genome ID: 648028043, Gene ID: 648047413), which is found outside of this clade, has an insertion and also contains a short cysteine rich C-terminal tail with the CX(3)CC grouping but lacks a full motif. *M. curtsii* also contains a duplicate ferrochelatase (Genome ID: 648047412), which has neither an internal insertion nor a C-terminal tail. The C-terminal MCFs, although being mostly grouped within distinct clades, are more widely distributed across the tree. This wide distribution of the C-terminal motif is also mirrored by its distribution within the phyla and may indicate loss of the cluster over time.

The presence of the [2Fe-2S] cluster coordinating residues has little impact on the evolutionary relationship of ferrochelatase. To assess the evolutionary relationship of ferrochelatase without the cluster coordinating residues, all internal insertion sequences and the C-terminal tails (regardless of presence of motif) were removed from the ferrochelatase sequences, and the remaining core sequences were aligned. The resulting tree (Fig. S1) is highly similar to the tree of the complete sequences, with the exception of the eukaryotic clade. In the tree lacking the motifs, the eukaryotic clade switches position with the firmicute clade, placing it closer to the Actinobacteria and Planctomycetes. This places the firmicute clade closest to the root in this model, potentially indicating that the firmicute ferrochelatase has changed the least over time compared to other ferrochelatases. It needs to be noted that not all eukaryotes were presented in the aforementioned clades. The plant ferrochelatases of *Z. mays* are found within a clade alongside the cyanobacteria that contain the CAB domain. The ferrochelatases of *P. falciparum* and *Leishmania major* are found within a clade that is made up predominantly of proteobacteria, some actinobacteria, and some firmicutes. It has been demonstrated that the parasite *Brugia malayi*, which lacks the heme synthesis pathway, obtained a functional and essential ferrochelatase through lateral gene transfer from an α-proteobacterial ancestor (38). It is likely that the two aforementioned parasites obtained their ferrochelatase in the same manner; however, this was not thoroughly assessed in this study. The sequences of the motifs alone were also aligned and assessed (Fig. S2). However, due to the short length and significant sequence variation, no distinguishable relationships could be identified.

### Physiological correlations

Given the scattered and somewhat sporadic distribution of cluster motifs, it was of interest to see if the presence of a cluster motif in ferrochelatase correlated with any physiological property. Toward this end a number of attributes were assessed: GC content, gene neighborhood, environment, oxygen requirement, and growth temperature. Metabolism and motility were also originally considered; however, too little data were available for these criteria to make an accurate assessment.

**Figure 6:**
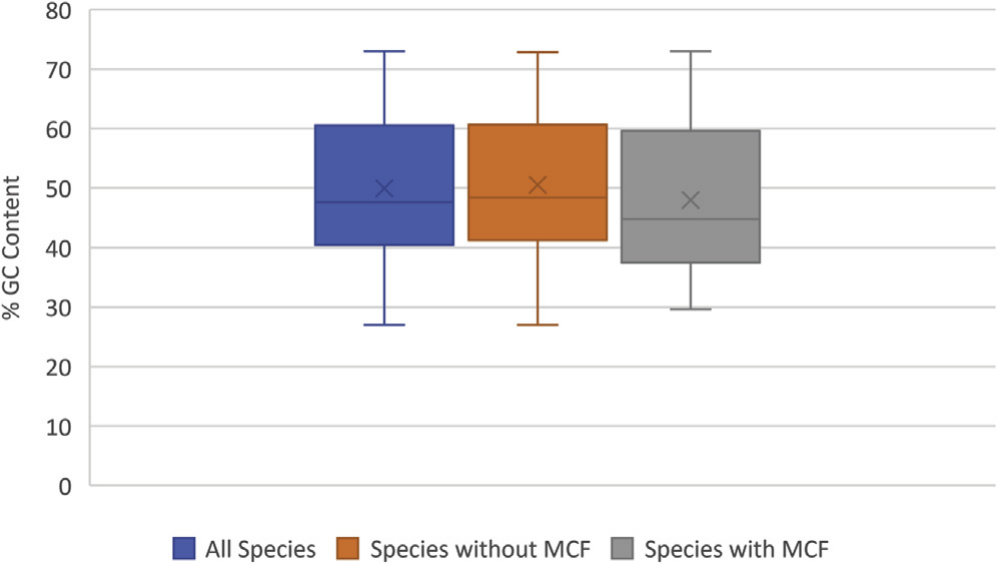
**GC content of sampled species.** A box and whiskers representation of the of GC content of sampled species. All species represented in *blue*, species containing sequences with a motif in *orange*, and species with sequences lacking a motif in *gray*. The X in each box denotes the arithmetic mean.

The average GC content of all assessed species is 50%, and the average of species with nonmotif ferrochelatase is 51% (Fig. 6). The average GC content of species containing MCFs is lower at 48%. Species with MCFs have a lower average GC content due to the low GC content of species carrying ferrochelatase with the internal insertion (average GC content of 45%). The Bacteroidetes, which contain the majority of insertion motifs, have a low GC content of 41%. The species containing C-terminal MCFs have an average GC content of 56%, which is not surprising due to its scattered distribution throughout multiple phyla. Although the average GC content of MCF containing species is somewhat lower, it is unlikely that it is tied to the presence of the motif, but rather due to its phylogenetic distribution. Img.JGI was used to assess the gene neighborhoods of the MCFs. However, no pattern was detected between ferrochelatase-containing neighborhoods with or without motifs.

Analysis did relieve that MCF-containing species are most commonly associated with soil and aquatic environments (Table 2). Overall, 42% of all MCF containing species were isolated from aquatic environments and 26% were isolated from soil. On the other hand, MCF-containing species are less associated with a host, with only 18% of MCF-containing species being host associated. However, there is no distinct pattern to type of host associated with MCF containing species. As the majority of species with MCF are Bacteroidetes, this may be a reflection of their typical environment.

**Table 2:** Environment of ferrochelatase-containing species

Environment	Species	MCF	% of total MCF species
Aquatic			
Freshwater	69	18	20%
Marine/salt water	57	15	17%
Hot spring/thermal vent	24	3	3%
Undefined	10	1	1%
Terrestrial			
Soil	71	23	26%
Geological	4	0	0%
Host			
Human	29	2	2%
Bovine	6	0	0%
Plants	11	3	3%
Other	35	11	13%
Industry			
Acid mine drainage	4	0	0%
Wastewater/sludge/sewage	7	1	1%
Food	7	1	1%
Other	8	0	0%
Other	7	2	2%

The majority of MCF-containing species identified in this analysis are mesophiles (Table 3). This is not surprising as 72% of all sequences assessed are mesophiles. What is of note is that only a small fraction of thermophiles (8%) contain MCFs, and none of the Hyperthermophiles assessed contain MCFs.

**Table 3:** Motif frequency by growth temperature

Growth Temp	Number of species	
	Total	Containing MCF
Psychrophile	9	3
Psychrotolerant	3	2
Mesophile	300	71
Thermophile	49	4
Hyperthermophile	4	0
No data	49	8

The majority of MCF-containing species grow aerobically (Tables 4 and 5). Note that this may also include facultative growth as data about anaerobic growth/fermentation was not available for “aerobic” described species. A total of 72% of species with MCFs are either aerobic or strictly aerobic. This is not necessarily surprising as 44% of assessed species have been described as aerobic. [2Fe-2S] clusters are often associated with anaerobic metabolism and are usually oxygen-labile (which has been noted for *H. sapiens* ferrochelatase) (37). Thus, it was somewhat unexpected that so few of the MCF-containing species are anaerobic and facultative anaerobic, specifically 7% of facultative anaerobic species and 5% of anaerobic species contain MCFs. As a result, the presence of MCF was compared to the presence of the [2Fe-2S] cluster ferredoxins (fer2 [2Fe-2S] cluster binding domain superfamily, COG0633) within the sampled species. The distribution of ferredoxin containing species by oxygen requirement is similar to that of species with MCF. This suggests that anaerobes do not necessarily have a high abundance of [2Fe-2S] cluster containing proteins. Additionally, there are more ferredoxin-containing species than there are species with MCFs. This suggests that the presence of other [2Fe-2S] cluster proteins does not indicate a [2Fe-2S] containing ferrochelatase within the same species.

**Table 4:** Oxygen requirement of MCF-containing species

Oxygen requirement	Number of species	
	Total	Containing MCF
Strictly aerobic	32	14
Aerobic	181	49
Microaerophilic	20	4
Facultative	55	4
Anaerobic	57	3
Strictly anaerobic	9	4
No data	60	10

**Table 5:** Ferredoxin (COG0633) and MCF distribution by oxygen requirement

Oxygen requirement	No MCF or COG0633	COG0633 only	MCF only	Both MCF and COG0633
Strict aerobic	5	13	2	12
Aerobic	4	128	45	4
Facultative	16	35	3	1
Microaerophilic	11	5	3	1
Anaerobic	36	18	3	0
Obligate anaerobic	2	3	4	0
No data	14	45	1	0

## Discussion

Since its discovery in 1994, the [2Fe-2S] cluster in ferrochelatase has proven to be an interesting enigma. It is clear that the presence of the [2Fe-2S] motif is not highly prevalent across all phyla with only 21% of the sampled genomes containing MCFs. However, the distribution of these few motifs within the phyla suggests that the cluster performs a function that provides a selective advantage to those organisms possessing it. To date, the metazoan ferrochelatase [2Fe-2S] cluster is the most characterized and has provided some functional insights, whereas characterized microbial MCFs have not provided any clues toward its function for the [2Fe-2S] cluster. From a structural standpoint the geometry of coordination of the [2Fe-2S] cluster in the human enzyme is unique. In characterized nonferrochelatase [2Fe-2S] cluster-containing proteins, the side chains of the four coordinating cysteines typically lie parallel to the plane of the cluster, whereas in human ferrochelatase, the side chains are almost perpendicular to the plane of the cluster (39). While the cluster in metazoan ferrochelatases is poorly stable, the clusters found in bacteria are incredibly robust, although it is likely that the cysteine geometry is similar between the two domains. From a functional standpoint, data for the metazoan enzyme show that the cluster is sensitive to mitochondrial membrane potential and is destroyed by NO both *in vitro* and *in vivo* (21, 22). Additionally it has been suggested that the essential nature of the cluster for enzyme activity provides an iron regulatory point in heme synthesis (37). Despite these functional attributes of the metazoan ferrochelatase [2Fe-2S] cluster, they are not mimicked by the microbial ferrochelatase [2Fe-2S] cluster.

Although the function/purpose of the microbial ferrochelatase [2Fe-2S] cluster has yet to be determined, the fact that it is prevalent in a subset of microbes suggests that it plays some significant role. Of interest is that within bacteria there exist two very distinct sets of coordinating cysteine residues, one located in an internal insertion and another at the carboxy terminus, yet the spatial positioning of the cluster within the three dimensional structure of the protein appears to be highly similar and adjacent to the active site pocket (Fig. 2). Perhaps this is a clue to its function. Nevertheless, even though the function of the cluster still eludes us, the phylogenetic distribution and environmental association of the cluster indicate points of interest.

The phylogenetic distribution of the [2Fe-2S] cluster motif (Fig. 4) suggests that the clusters (internal *versus* C-terminal) evolved independently of one another. The placement of Firmicute ferrochelatase closest to the root of the “core” sequence tree indicates that ferrochelatases likely acquired a C-terminal extension over time. It is possible that this C-terminal extension carried a [2Fe-2S] cluster, which subsisted in metazoans but was lost in most microbes.

The internal insertion, however, is more conserved within distinct phyla than the C-terminal motif, but was also likely acquired after the C-terminal extension. In total, 97% of sequences that contain the internal insertion carry a [2Fe-2S] motif, yet the insertion is found in only a handful of phyla. This conserved and prevalent nature of the insertion motif within such a small subset of phyla indicates an evolutionary/survival requirement for this motif within these species. The origin of the insertion could be due to a recombination event of the C-terminus into the center of the sequence that generated the divergence of the clades. Closer analysis of the Bacteroidetes phylum outliers provides some insight to the progression of these evolutionary events. Specifically, the two ferrochelatase sequences of *S. ruber*, where both lack the internal insertion, but one has the C-terminal motif, could be an example of a prerecombination sequence. The ferrochelatase of *P. gingivalis*, on the other hand, which contains the insertion but lacks the motif, may be an example of a species where the selective pressure for the cluster was lost, potentially due to similar environment as C-terminus containing species. It could be posited that a cluster-encoding motif in the center of the protein is preferred in a highly selective environment as it would ensure the cluster would be part of the final peptide even after premature translational termination (*e.g.*, due to Rho-dependent termination or near stop codons) (40). As such, ferrochelatase with an internal cluster may have a greater evolutionary advantage/requirement than those with a C-terminal cluster. Whereas those with a C-terminal extension no longer had the selective pressure to maintain the cluster, which is also supported by the fact that some C-terminal tails contain partial motifs due to deletions or substitutions.

The frequency and phylogenetic distribution of the eukaryotic MCFs support current evolutionary models of the tree of life. In eukaryotes, ferrochelatase is nuclear encoded, expressed in the cytosol, and translocated to the mitochondrion (3). Metazoan ferrochelatases contain [2Fe-2S] clusters coordinated by the C-terminal, suggesting that the original bacterial symbiont that evolved into the mitochondrion also possessed a C-terminal motif. However, despite containing a C-terminal extension, not all eukaryotic ferrochelatase contain a [2Fe-2S] cluster. To date, evolutionary studies have identified an Alphaproteobacterial ancestor to be the most likely candidate for the source of the mitochondrion (41). In total, 25% of the Alphaproteobacterial ferrochelatases assessed in this study contain C-terminal motifs. It is likely that the common mitochondrial ancestor contained a C-terminal extension with a [2Fe-2S] cluster but that some eukaryotes lost the cluster over time. It is also of note that the Firmicute ferrochelatases were designated as evolutionarily more ancient than the eukaryotic ferrochelatases, positing that firmicutes may be more ancient than the mitochondrial ancestor, a notion that has already been reported in the literature (42, 43).

When considering the evolution of the full motif, the presence/absence of the incomplete CX(x)CC motif that is missing the fourth cysteine needs to be noted. The majority of the ferrochelatase with the motif in the insertion and approximately 50% of the C-terminal motif have adjacent cysteines, *i.e.*, CX(x)**CC**X(x)C. However, the CX(x)CC grouping that is lacking the fourth cysteine is common in nonmotif-carrying ferrochelatases and while more commonly found in the C-terminal tail is also found in the internal insertion. Generally, adjacent cysteines in a protein sequence are not common but when present can be responsible for membrane anchoring through palmitoylation, loop formation, and stabilization through disulfide bridges, may act as a redox switch, and have shown to be part of [4Fe-4S] coordination (44, 45, 46, 47). It is possible that this grouping is a remnant of prior [2Fe-2S] motifs, where the [2Fe-2S] cluster was lost over time. However, when considering that the metazoan motif does not contain adjacent cysteines, it could be posited that the C-terminal motif had two lineages one with adjacent cysteines and one without.

Interestingly, the phylogenetic distribution may shed more light on the purpose of the [2Fe-2S] cluster when looking at the habitat and growth requirements of the MCF species. MCFs are significantly more common to aerobic mesophiles found in soil or aquatic environments (Table 2, Table 3, Table 4). The oxygen requirement of MCFs species on the surface may be somewhat surprising given that in general iron sulfur clusters are known for their oxygen lability (48, 49). However, the microbial ferrochelatase [2Fe-2S] clusters examined to date are quite stable in the presence of O_2_. The absence of the cluster in anaerobic species and its stability in oxidizing conditions suggest that the purpose of the cluster may be associated with aerobic metabolism. Considering the functions of the product, heme, in respiratory chains, peroxidases and catalases, and aerobic xenobiotic metabolism, having a redox sensitive function built into ferrochelatase seems reasonable. Soil and aquatic environments are exceedingly heterogeneous compared with host and industrial environments. This is especially the case when considering O_2_ availability; for example, wind and currents can increase or decrease at the depth at which a microbe is suspended, and rain increases soil density. It is therefore plausible that the [2Fe-2S] aids in adapting to these changes. Interestingly, *S. ruber* and *X. tropicalis* contain duplicate ferrochelatases with one possessing a cluster motif and the other not. It will be of interest to learn if their expression is environmentally regulated/sensitive.

Overall, the low percentage of MCF found in thermophilic species (Table 3) was somewhat surprising. Iron sulfur clusters are typically heat-stable and have been associated with a number of thermophiles and hyperthermophiles, so there is no direct temperature-related reason why thermophiles would not possess a ferrochelatase with a [2Fe-2S] cluster (50, 51). In addition, 70% of thermophiles sampled are aerobic and some are from phyla that contain species with MCFs, such as Bacteroidetes, Actinobacteria, and Proteobacteria. Thus, there are no obvious explanations as to why thermophilic species possess ferrochelatases without a [2Fe-2S] cluster. Conversely, the majority of psychrophilic species sampled contain a MCF. Therefore, MCF sequences are more commonly associated with colder rather than warmer habitats.

When taking this data into consideration, some pitfalls need to be noted. The majority of this work relies on correct annotation of the selected genomes. With the relative few clades that have characterized ferrochelatase, and the significant increase of published genomes, misannotation of genes is not uncommon and could have implications on this study (52). Although each sequence was vetted for the presence of the catalytic histidine, it is possible that the data set contains isozymes that no longer function as ferrochelatase or that some functional ferrochelatases were eliminated (taking the case of *B. cinerea* as an example). In addition, the emphasis on sequencing species with human centric purposes has skewed the available data toward a handful of phyla and environmental data are incomplete, as several of the species sampled have not been cultured yet.

Importantly, this study sheds light on the distribution of [2Fe-2S] motifs in ferrochelatases. These data have not only identified potential [2Fe-2S] carrying ferrochelatase but will also aid in assessing the likelihood of a ferrochelatase sequence containing a motif. In addition, this study has discovered potential links between the presence of the [2Fe-2S] cluster, aerobic metabolism, and cooler aquatic and soil environments. These links will be helpful in identifying the role of the ferrochelatase [2Fe-2S] cluster.

## Experimental procedures

### Sequence Selection

Ferrochelatase sequences were acquired through Integrated Microbial Genomes Expert Review (IMG/MER) portal (https://img.jgi.doe.gov/) on 09/18/2020 (26, 27). Ferrochelatase containing genomes were identified from “all isolates” using the “Find Function” search, with the Enzyme I.D. E.C. 4.99.1.1 and “Finished” sequencing status as search parameters. Archaeal genomes were removed from the resulting list. To pare down the genomes, one genome per genus was selected manually (preference was given to type strains and genomes with characterized ferrochelatase). The list was further compressed by assessing phylogenetic distribution and selecting ten genomes or fewer per order, ensuring that the distribution was equal across the families (Table S2). The resulting genome table was then redisplayed with the following information and exported to Excel (Table S1): Genome ID, Genome/Sample Name, Domain, Phylum, Class, Order, Family, Species, Energy Source, Geographic Location, Metabolism, Oxygen Requirement, pH, Salinity, Temperature Range, Habitat, Motility, and GC∗ Assembled. Ferrochelatase genes were selected by searching for the ferrochelatase enzyme function (E.C. 4.99.1.1) within the selected genomes. Identified genes were then exported as translated amino acid text files.

### Sequence alignment

The exported gene file was imported into Geneious Prime (v. 2019.2.1) for sequence alignment and generation of a phylogenetic tree. The sequences were aligned using MUSCLE alignment, with a maximum of eight iterations (53). The sequences were assessed for the presence of the essential catalytic histidine (H263 human ferrochelatase numbering) and sequences missing this residue were excluded from the alignment. Sequence segments that resulted in gaps in more than 95% of the sequences were removed manually, and the sequences were realigned using the same method as mentioned earlier. The resulting alignment was assessed for gaps and realigned for a second time, resulting in the final alignment (Fig. S1). The final alignment was used to identify sequences containing the N-terminal extension, the internal insertion, the C-terminal tail, and the motif.

### Generation of consensus tree

The final alignment was used to generate the consensus tree. The tree was generated using the Geneious consensus tree builder with the following constraints: Jukes Cantor Genetic Distancing Model, Neighbor Adjoining Tree Build Method, No Outgroup, Bootstrap Resampling Method, Random Seed 95,549, 100 Replicates, 50% Support Threshold, and 0% Topology Threshold. The resulting Tree was annotated using the EMBL Interactive Tree of Life online server (54).

### Assessment of species and sequence properties

Genomes that contained an enzyme lacking the catalytic histidine (assessed in first alignment) were removed from genome table (with the exception of genomes that carried a duplicate functional ferrochelatase). The genome table was assessed for completeness. Empty cells, lacking information, were researched, and filled in manually. The resulting genome information was linked to the final list of ferrochelatase sequences by the Genome ID. Cysteine counts for all ferrochelatase sequences were assessed using Pymol and added to the final Excel genome/gene table. With the exception of the binary logistical regression, all analyses were performed using standard Excel functions. The Binary Logistical Regression was performed using IBM SPSS (v. 26) statistical software.

### Identification of ferredoxin containing species

In IMG/MER, the Function search tool was used to search for the clusters of orthologous groups of proteins (COG) identifier 0633, within the final list of 414 genomes (55, 56). In total, 273 genomes were identified to encode one or more ferredoxin proteins. The Genome IDs were exported to Excel and compared with the existing genome table.

## Data availability

Microbial genomes and physiological data are available through the IMG.JGI website (https://img.jgi.doe.gov/). Further information on bacterial physiological data can be found on the BacDive website (https://bacdive.dsmz.de/). A tree of life and a deposition of the trees created for this study can be found at the Interactive Tree of Life Website (https://itol.embl.de/shared/1YFbOwXl23wtM).

## Supporting information

This article contains supporting information.

## Conflict of interest

The authors declare that they have no conflicts of interest with the contents of this article.
