# Supplementary material for: Ironing out the distribution of [2Fe-2S] motifs in ferrochelatases
Source: J Biol Chem. 2021 Sep 25;297(5):101017. doi: 10.1016/j.jbc.2021.101017 (PMC8529089; doi:10.1016/j.jbc.2021.101017)
Supplement: Figures S1 and S2 [file mmc1.docx]

**Supporting Information**

**
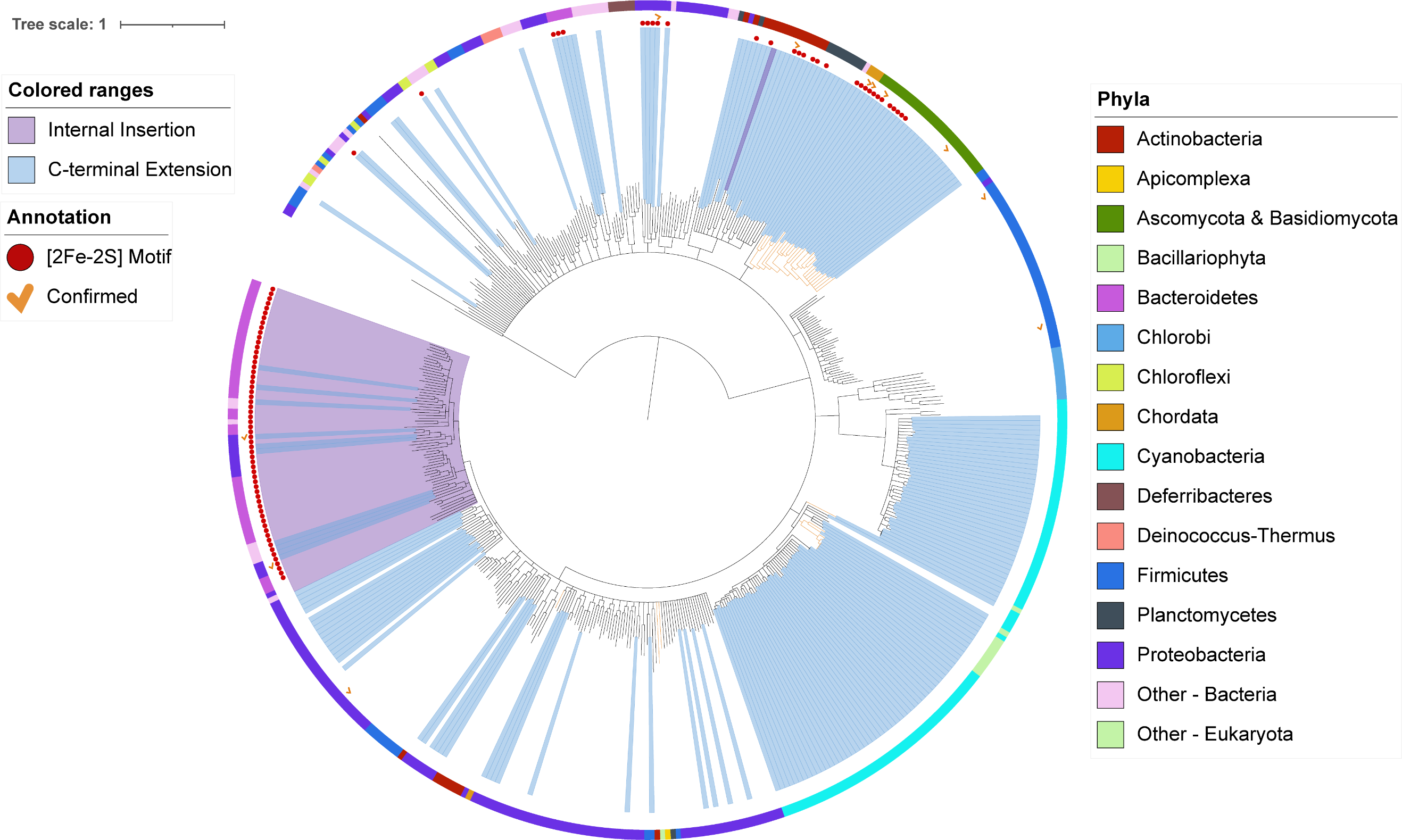
**

**Supplemental Figure 1. Phylogenetic Tree of Ferrochelatases, Internal Insertion and C-terminal Extensions Removed.**

Black lines indicate bacterial species and orange-colored lines indicate eukaryotic species. Colored bars indicate their phylum (see Phyla legend). The icons indicate the presence of the [2Fe-2S] Motif (red dot), and whether the enzyme has been characterized (orange checkmark). The interactive version of the trees can be accessed at <https://itol.embl.de/shared/1YFbOwXl23wtM> .

**
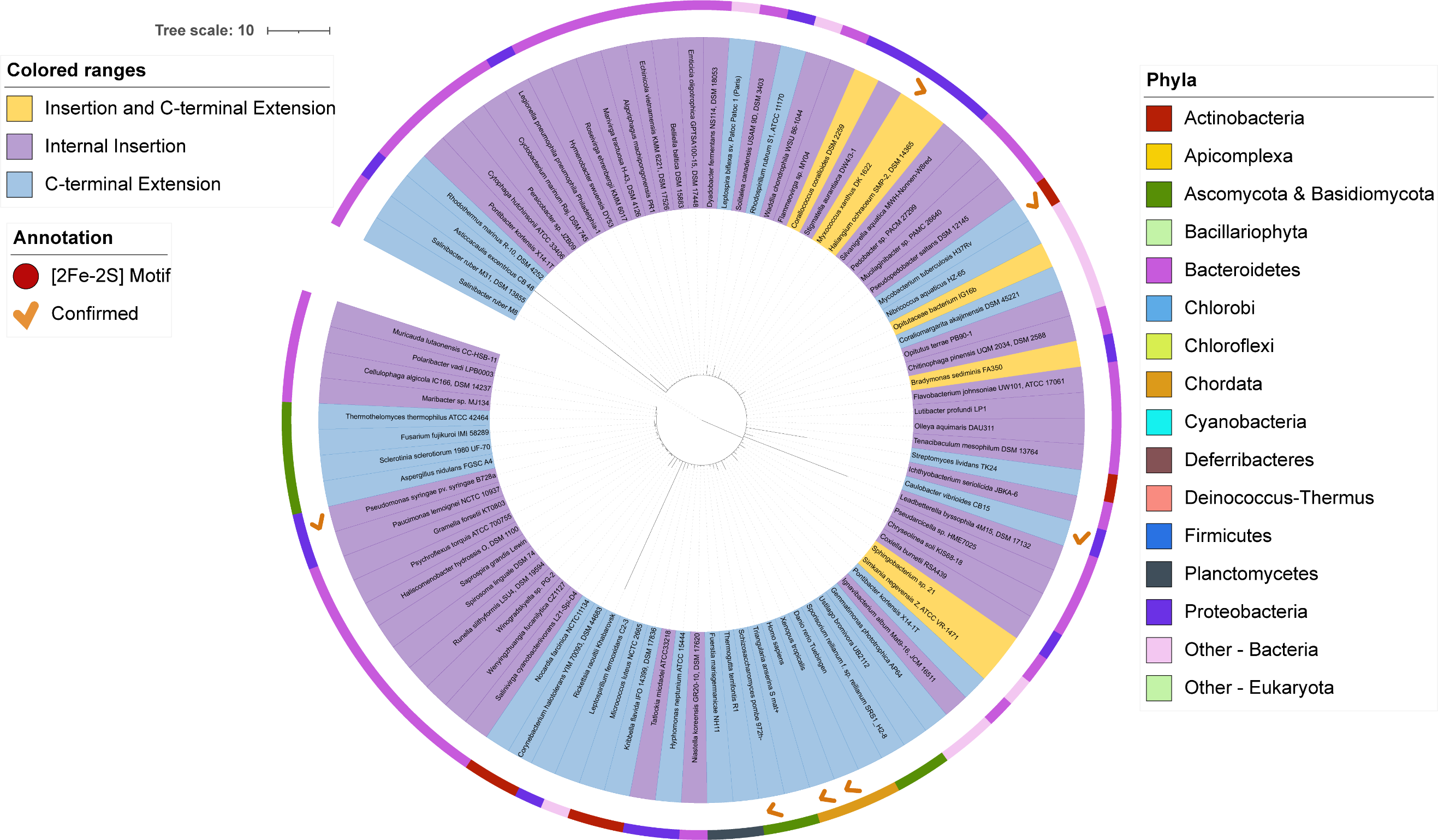
**

**Supplemental Figure 2. Phylogenetic Tree of Ferrochelatases, Motifs Only**

Black lines indicate bacterial species and orange-colored lines indicate eukaryotic species. Colored bars indicate their phylum (see Phyla legend). The icons indicate the presence of the [2Fe-2S] Motif (red dot), and whether the enzyme has been characterized (orange checkmark). The interactive version of the trees can be accessed at <https://itol.embl.de/shared/1YFbOwXl23wtM> .
